# Robust Fast 3D Beam Alignment for UAV-Assisted mmWave and Terahertz Communications

**DOI:** 10.3390/s26113612

**Published:** 2026-06-05

**Authors:** Loubna Gafari, Wissal Attaoui, Essaid Sabir, Elmahdi Driouch

**Affiliations:** 1 NEST Research Group, LRI Laboratory, École Nationale Supérieure d’Électricité et de Mécanique (ENSEM), Hassan II University of Casablanca, Casablanca 20000, Morocco; 2 École Nationale Supérieure d’Informatique et d’Analyse des Systèmes (ENSIAS), Mohammed V University of Rabat, Rabat 10056, Morocco; wissal.attaoui@ensias.um5.ac.ma; 3 Department of Science and Technology, TÉLUQ, Université du Québec, Montreal, QC H2S 3L4, Canada; 4 Department of Computer Science, University of Quebec at Montreal (UQAM), Montreal, QC H2L 2C4, Canada; driouch.elmahdi@uqam.ca

**Keywords:** UAV communications, mmWave, terahertz, initial access, beam alignment, stochastic geometry, URLLC, HRLLC, risk-aware optimization, latency variance

## Abstract

Unmanned aerial vehicle (UAV)-assisted millimeter-wave (mmWave) and terahertz (THz) communications are promising enablers of ultra-reliable and low-latency communication in next-generation wireless networks. However, the initial access and beam alignment process remains challenging because highly directional beams must be rapidly aligned in a three-dimensional environment. In this paper, we investigate a risk-aware beam alignment framework for UAV-assisted mmWave/THz systems, where user equipment scans a 3D spherical region to detect UAV base stations. The objective is to jointly minimize the expected cell-search latency and its variance while satisfying detection-failure and link-quality constraints. To solve this non-convex optimization problem efficiently, we employ the Lévy Self-Renewable Flow Direction Algorithm (LSRFDA), which combines Lévy-flight exploration with self-renewal to improve convergence robustness. A unified propagation model is adopted to cover both mmWave and THz regimes by incorporating free-space spreading loss and frequency-dependent molecular absorption. Extensive Monte Carlo simulations compare the proposed approach with Particle Swarm Optimization, Random Search, Reinforcement Learning, and PPO-Lagrangian methods. The results show that LSRFDA achieves lower latency, lower latency variation, more reliable detection, and lower energy consumption across a wide range of UAV densities and coverage radii. These outcomes highlight the effectiveness of risk-aware geometric optimization for fast and dependable initial access in UAV-assisted 5G mmWave and 6G THz networks.

## 1. Introduction

5G Ultra-Reliable Low-Latency Communication (URLLC) and 6G Hyper-Reliable Low-Latency Communication (HRLLC) are two key requirements for many emerging wireless applications, such as autonomous systems, industrial automation, remote control, and emergency services. In these scenarios, the network must provide both very low delay and high reliability. Unmanned Aerial Vehicles (UAVs) have recently attracted strong interest as aerial base stations because they can be deployed quickly and can provide flexible wireless coverage in dynamic environments [1,2,3].

Millimeter-Wave (mmWave) and Terahertz (THz) frequency bands are promising for future wireless networks because they offer very large bandwidths and can support very high data rates. However, communication at such high frequencies experiences severe propagation loss. To compensate for this effect, both transmitters and receivers rely on highly directional beams. Because of this strong directionality, the initial access procedure becomes a critical part of the communication process. Before data transmission starts, the user equipment (UE) must detect a nearby base station and align its beam toward it. In UAV-assisted networks, this task is more difficult because communication takes place in a three-dimensional (3D) space, where both the UE and the UAV may have different locations and orientations. During the initial access phase, the UE scans the surrounding space using directional beams with a given half-power beamwidth (HPBW). The choice of beamwidth has a direct effect on the search process. A wide beam covers a larger area and may reduce the number of scanning steps, but it provides lower directional gain. By contrast, a narrow beam offers higher gain and can improve link quality, but it requires more scanning directions and may increase access delay. Therefore, beamwidth selection creates a trade-off between search speed and communication quality [4,5].

### 1.1. Related Work

Recent studies have investigated the use of UAVs as aerial base stations to support URLLC services in mmWave networks. In [6], the authors analyzed beam alignment and cell-search challenges in UAV-enabled mmWave systems, highlighting the impact of mobility and blockage. Refs. [7,8] provides a comprehensive overview of initial access and beam management techniques in mmWave and terahertz systems, discussing the trade-offs latency, energy efficiency, and reliability.

More recent works have focused on improving beam alignment efficiency in dynamic 3D environments. Stochastic geometry and spatial modeling tools have been widely used to characterize detection probability and optimize initial access procedures [8,9]. In parallel, several studies have addressed the non-convex nature of beamwidth selection using metaheuristic optimization methods, including PSO and related approaches [10,11]. Moreover, learning-based beam alignment strategies have gained increasing attention. Reinforcement learning and hybrid search techniques have been applied to adapt beam directions in UAV-assisted mmWave and THz systems [12,13]. More recently, learning-assisted beam alignment and geometric approaches have been proposed to reduce search complexity and improve alignment accuracy in highly directional systems [14]. These works show that combining spatial modeling with adaptive search can significantly improve beam alignment performance.

Hybrid mmWave and THz communication schemes have also been proposed to balance data rate and reliability, especially in URLLC scenarios [15]. In parallel, research has focused on UAV-based URLLC, particularly their deployment flexibility and link-layer adaptation [16]. These works confirm that directional beam alignment remains a key step in ensuring stable and efficient communication. At the same time, a number of recent contributions have explored beam alignment strategies that rely on geometric models or adaptive search methods. Some approaches aim to reduce the number of beam directions to be scanned, while others try to improve detection accuracy by combining directional gain with learning or sensing information. Despite these efforts, most existing methods still focus mainly on average latency or detection probability.

Only a limited number of studies have considered more stringent reliability metrics, such as latency variation or tail behavior, which are important for URLLC services [17,18]. In most cases, the analysis is limited to average performance, without explicitly accounting for fluctuations that may affect time-critical applications. Although several recent works have improved beam alignment through learning-based or heuristic approaches, they often require extensive training or rely on complex parameter tuning. This can limit their practical use in fast-changing UAV environments, where rapid adaptation is required.

Recent work has also explored Integrated Sensing, Communication, and Computing (ISCC) in UAV-assisted networks, where beam management and latency-aware access stand out as critical design factors. Incentive-based scheduling under ISCC constraints is studied in [19], where resource competition directly constrains how frequently beam alignment can be triggered. A UAV-RIS-assisted covert ISCC system for near-field low-latency operation is presented in [20], highlighting the overhead that reliability constraints impose on initial access. Security constraints in UAV-aided ISCC systems are examined in [21], where authentication overhead adds a non-negligible contribution to latency variance. Joint resource and trajectory optimisation under ISCC settings is addressed in [22], underscoring the importance of robust initial access in these integrated systems. The above works collectively reveal a gap that remains largely unaddressed: while latency and reliability are recognised as critical in UAV-assisted networks, existing methods mainly optimise average performance metrics, leaving latency variance uncontrolled. In fast-changing 3D environments, this is insufficient for URLLC and HRLLC services, where worst-case delay matters as much as the mean. To address this, the initial access problem is formulated here as a risk-aware optimisation framework that jointly minimises both the expected cell-search latency and its variance. A lightweight metaheuristic solver, LSRFDA, is employed to find the optimal beamwidth without any training overhead, making the approach directly deployable in dynamic UAV environments.

Looking further ahead, the evolution of aerial communications toward 6G low-altitude aerial intelligent network (LAIN) architectures introduces highly dynamic multi-platform environments in which unified channel-model-driven optimisation frameworks become increasingly important for maintaining robust link reliability and scalable beam management [23].

### 1.2. Our Contribution

For URLLC/HRLLC systems, it is insufficient to consider only the average access delay. Delay variation is equally critical, since large fluctuations can reduce the reliability of time-sensitive services. Motivated by this gap, a beam alignment problem is formulated as a risk-aware optimisation framework that jointly considers both the mean and the variability of cell-search latency in UAV-assisted mmWave and THz networks.

The main contributions and insights of this work are as follows:

- A 3D stochastic-geometry framework is developed that quantifies both the mean and the variance of cell-search latency in UAV-assisted mmWave/THz networks under a Poisson point process (PPP) spatial model.
- A formal risk-aware beam alignment problem is formulated that jointly minimises expected latency and latency variance. It is shown through simulation that the variance-optimal beamwidth differs from the average-latency-optimal beamwidth, exposing a fundamental trade-off that is invisible in conventional average-based formulations.
- Simulation evidence is provided that the variance constraint (17b) is frequently binding at low UAV densities ($\lambda <10^{-3}$ m^−3^), demonstrating that risk-aware design is most valuable in the challenging sparse-deployment regime relevant to URLLC/HRLLC.
- The LSRFDA metaheuristic solver is employed, which exploits Lévy-flight exploration and periodic self-renewal to converge on the risk-aware optimal beamwidth without any training procedure, a key practical advantage over RL-based alternatives.
- A unified propagation model is adopted that captures both mmWave and THz attenuation effects (free-space spreading and molecular absorption), enabling cross-band analysis within a single framework.
- A comprehensive comparative evaluation is conducted against PSO, Random Search, RL (REINFORCE), and PPO-Lagrangian, covering a wide range of UAV densities, coverage radii, and propagation conditions.

The rest of this paper is organised as follows. Section 2 presents the system and channel models. Section 3 describes the optimisation problem and the proposed solution method, including the MDP formulation for DRL baselines and practical deployment considerations. Section 4 discusses the simulation results. Section 5 concludes the paper.

## 2. Problem Formulation

### 2.1. System Model

This section describes the considered network topology, key assumptions, and system operation during the initial access phase in UAV-assisted mmWave/THz networks.

#### 2.1.1. 3D Spatial Deployment

We consider a three-dimensional region where unmanned aerial vehicles operate as aerial base stations to support ground user equipment in mmWave or THz bands. UAVs are spatially distributed according to a homogeneous Poisson point process (PPP) with density λ (UAVs/m^3^). Both UAVs and users are equipped with planar antenna arrays whose half-power beamwidth θ defines the angular coverage of each beam. At a given time, UAVs and users are independently positioned within the coverage volume of radius *R*. This volume corresponds to a 3D sphere of volume $\frac{4}{3}\pi R^{3}$, consistent with the PPP modeling framework.

#### 2.1.2. UAV Mobility

UAV motion during the initial access interval is modeled as a bounded displacement $\Delta r\leq v_{\max}T_{\mathrm{scan}}\left(\theta \right)$, where $v_{\max}$ is the UAV maximum speed. Since the full scanning duration $T_{\mathrm{scan}}$ is typically on the order of milliseconds, the displacement remains negligible compared to *R*, validating the quasi-static approximation.

#### 2.1.3. 3D Beam Scanning

**Figure 1:**
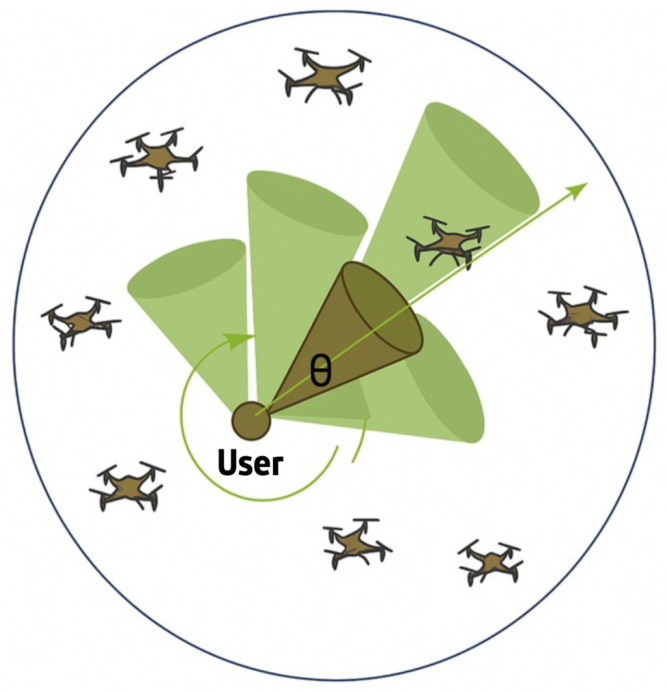
Spherical beam scanning model in the considered UAV-assisted mmWave/THz system. The user equipment (UE) sequentially sweeps a directional conical beam with half-power beamwidth $\theta_{\mathrm{UE}}$ over the three-dimensional angular space to detect UAVs located within a spherical coverage region of radius *R*.

The spherical beam scanning model is illustrated in Figure 1. During access, each UE periodically scans its surrounding space to locate UAVs. The conical beam of width θ subtends a solid angle

(1) $$\Omega_{\mathrm{beam}}\left(\theta \right)=2\pi \left(1-\cos(\theta /2)\right).$$

Full 3D coverage of the unit sphere therefore requires approximately

(2) $$N_{\mathrm{dir}}\left(\theta \right)=\left\lceil \frac{4\pi }{\Omega_{\mathrm{beam}}\left(\theta \right)}\right\rceil =\left\lceil \frac{2}{1-\cos(\theta /2)}\right\rceil .$$

For narrow beams, $N_{\mathrm{dir}}\left(\theta \right)\approx 16/\theta^{2}$, confirming the quadratic growth of scanning complexity in 3D.

#### 2.1.4. Scan-Time Model

The per-direction mini-slot includes beam switching, pilot estimation, processing, and a propagation bound 2R/c. The overall time per direction is

(3) $$t=t_{\mathrm{switch}}+t_{\mathrm{est}}+t_{\mathrm{proc}}+\frac{2R}{c}.$$

Hence, the total time required to sweep all directions is

(4) $$T_{\mathrm{full}}\left(\theta ,R\right)=N_{\mathrm{dir}}\left(\theta \right)\;t=\left\lceil \frac{2}{1-\cos(\theta /2)}\right\rceil \left(t_{\mathrm{switch}}+t_{\mathrm{est}}+t_{\mathrm{proc}}+\frac{2R}{c}\right).$$

#### 2.1.5. Access Operation

**Figure 2:**
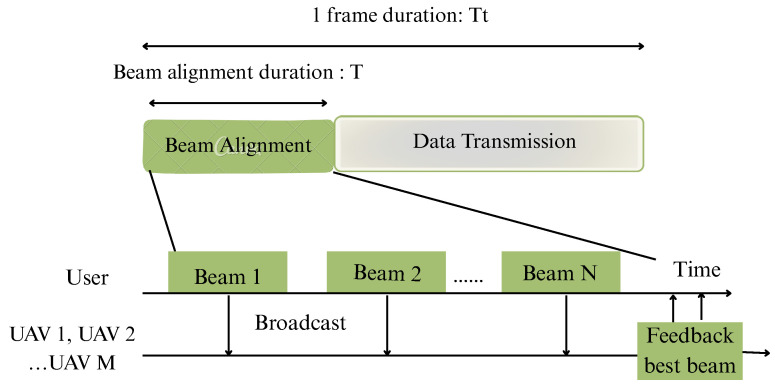
Illustration of the initial beam alignment and subsequent data transmission process in a UAV-assisted mmWave/THz communication system. The user equipment (UE) performs directional beam scanning to detect line-of-sight (LoS) UAV links, followed by beam alignment and data transmission.

At the beginning of the cell search, the UE transmits probing signals sequentially over all beam directions, as illustrated in Figure 2, attempting to detect any UAV whose received signal-to-noise ratio exceeds the threshold T. Successful detection initiates beam alignment and data exchange.

### 2.2. Transmission Model

#### 2.2.1. Directional Antenna Model

Each antenna is modeled using a simplified sectorized radiation pattern. The gain $G_{i}$ for node i∈{UAV,UE} depends on the beamwidth $\theta_{i}$ as

(5) $$G_{i}\left(\theta_{i}\right)=\left\{\begin{array}{cc} \frac{2\pi }{\theta_{i}}, & \mathrm{main}\;\mathrm{lobe},\\ \eta , & \mathrm{side}\;\mathrm{lobe}, \end{array}\right.$$

where η=−10 dB (0.1 in linear scale) denotes the side-lobe gain.

Both UE and UAV sequentially explore different beam directions to establish a line-of-sight connection. In the three-dimensional case, the number of steering directions follows from the solid-angle coverage of each conical beam,

(6) $$N_{i}\left(\theta_{i}\right)=\left\lceil \frac{2}{1-\cos(\theta_{i}/2)}\right\rceil .$$

#### 2.2.2. Received Signal-to-Noise Ratio

The received SNR at the typical user is given by

(7) $$\mathrm{SNR}=\frac{P_{\mathrm{UAV}}G_{\mathrm{UAV}}h_{\mathrm{UAV}}L_{\mathrm{UAV}}\left(d\right)\;S}{\sigma^{2}}=\frac{P_{\mathrm{UAV}}\frac{2\pi }{\theta }h_{\mathrm{UAV}}K^{2}d^{-\alpha }}{\sigma^{2}}=\frac{\frac{2\pi }{\theta }h_{\mathrm{UAV}}d^{-\alpha }}{\sigma_{m}^{2}},$$

where $L_{\mathrm{UAV}}\left(d\right)=K^{2}d^{-\alpha }$ models large-scale path loss, *d* is the UAV–UE separation distance, α is the path loss exponent, and $K=\frac{c}{4\pi f_{m}}$ is the frequency-dependent attenuation constant.

#### 2.2.3. LoS Probability and Blockage

The probability that a UAV–UE link remains in line-of-sight condition decays exponentially with distance according to

(8) $$\mathrm{Pr}\left(S=1\right)=e^{-\beta r},$$

where β is an environment-dependent blockage parameter [24].

### 2.3. Energy Consumption Model

The total energy consumed during the beam alignment process is defined as:

(9) $$E=P_{\mathrm{UE}}\cdot T_{\mathrm{scan}}\left(\theta \right),$$

where $P_{\mathrm{UE}}$ is the UE transmit power and $T_{\mathrm{scan}}\left(\theta \right)$ is the total scanning duration. Since $T_{\mathrm{scan}}\left(\theta \right)=N_{\mathrm{dir}}\left(\theta \right)\cdot t$, the energy consumption becomes:

(10) $$E=P_{\mathrm{UE}}\cdot N_{\mathrm{dir}}\left(\theta \right)\cdot t.$$

### 2.4. Performance Metrics

#### 2.4.1. Successful Detection Probability

The probability that at least one UAV is successfully detected within the scanned beam region is expressed as

(11) $$P_{s}\left(\theta \right)=1-\exp\;\left(-\lambda \;V_{\sec}\left(\theta ,R\right)\;P_{\mathrm{LoS}}\;P_{\mathrm{SNR}}\right),$$

where $V_{\sec}\left(\theta ,R\right)$ denotes the volume of the 3D spherical sector covered by the UE beam, $P_{\mathrm{LoS}}=\exp\left(-\beta r\right)$ is the line-of-sight probability, and $P_{\mathrm{SNR}}=\mathrm{Pr}\left(\mathrm{SNR}\geq T\right)$ represents the probability that the received SNR exceeds the detection threshold. The spherical-sector volume is given by

(12) $$V_{\sec}\left(\theta ,R\right)=\frac{2\pi R^{3}}{3}\left(1-\cos\frac{\theta }{2}\right).$$

#### 2.4.2. Detection Failure Probability

The probability that no UAV is detected after scanning $N_{c}$ mini-slots is

(13) $$P_{f}\left(N_{c}\right)=\max\left({\left(1-P_{s}\right)}^{N_{c}},\;P_{\mathrm{no}-\mathrm{LoS}}\right),$$

where

(14) $$P_{\mathrm{no}-\mathrm{LoS}}=\exp\left(-\frac{2\lambda \pi }{\beta }\right).$$

#### 2.4.3. Cell-Search Latency

The normalized expected latency is given by

(15) $$\frac{E[L\left(N_{c}\right)]}{t}=\frac{1-\left(N_{c}+1\right){\left(1-P_{s}\right)}^{N_{c}}+N_{c}{\left(1-P_{s}\right)}^{N_{c}+1}}{\left(1-P_{f}\right)P_{s}},$$

where *t* is the duration of a single mini-slot defined in (3).

**Remark 1.** 
*For any $N_{c}\in N$, the instantaneous cell-search latency satisfies*

(16) $$t_{0}\leq L\left(N_{c}\right)\leq N_{c}t,$$

*where $t_{0}$ is the minimum achievable detection time.*

## 3. Optimal Initial Beam Alignment

### 3.1. Risk-Aware Optimal Beam Alignment

Based on the above characterizations, the main goal is to select the user beamwidth $\theta_{\mathrm{UE}}$ to minimize cell-search latency under a detection failure probability constraint. The closed-form expression for the latency variance is derived in Appendix A. The optimization problem is formulated as

(17a) $$\begin{array}{cc} \min_{\theta }\; & \frac{E[L\left(N_{c}\right)]}{t}+\mathrm{Var}\left(L\left(N_{c}\right)\right) \end{array}$$

(17b) $$\begin{array}{cc} s.t.\;\; & \mathrm{Var}\left(L\left(N_{c}\right)\right)<{\mathrm{Var}}_{\max}, \end{array}$$

(17c) $$\begin{array}{cc} & P_{f}\left(N_{c}\right)\leq P_{f}^{\max}, \end{array}$$

(17d) $$\begin{array}{cc} & \max_{x_{i}}{\mathrm{SNR}}_{x_{i}}\geq T, \end{array}$$

(17e) $$\begin{array}{cc} & 0\leq \theta \leq \pi . \end{array}$$

This formulation explicitly balances performance reliability and temporal uncertainty, aligning with risk-aware design principles for URLLC/HRLLC systems.

**Remark 2** 
(Risk-Aware Insight)**.** *The solution to (17) differs fundamentally from the beamwidth that minimizes $E[L\left(N_{c}\right)]$ alone. Simulation results indicate that the risk-aware optimal beamwidth is generally narrower, and is observed to be approximately 10–30% smaller than the average-latency-optimal beamwidth across the considered UAV density range. Moreover, the variance constraint (17b) becomes active in sparse UAV deployment scenarios ($\lambda <10^{-3}$ m^−3^), demonstrating that optimization based solely on average latency may lead to violations of stringent URLLC tail-latency requirements in challenging network conditions.*

**Remark 3** 
(Risk-Aware Beamwidth Behaviour)**.** *For sparse UAV deployment regimes, the optimal beamwidth obtained from the proposed risk-aware formulation is generally narrower than the beamwidth that minimizes only the average latency, i.e.,*

(18) $$\theta_{\mathrm{risk}}^{*}<\theta_{\mathrm{mean}}^{*}.$$

*This behaviour arises because narrower beams provide higher directional gain and reduce latency fluctuations caused by intermittent detection failures in sparse 3D environments. Consequently, the variance constraint in Problem (17) becomes active at low UAV densities, forcing the optimization toward more reliable beam configurations.*

### 3.2. Risk-Aware Optimisation Framework Overview

**Figure 3:**
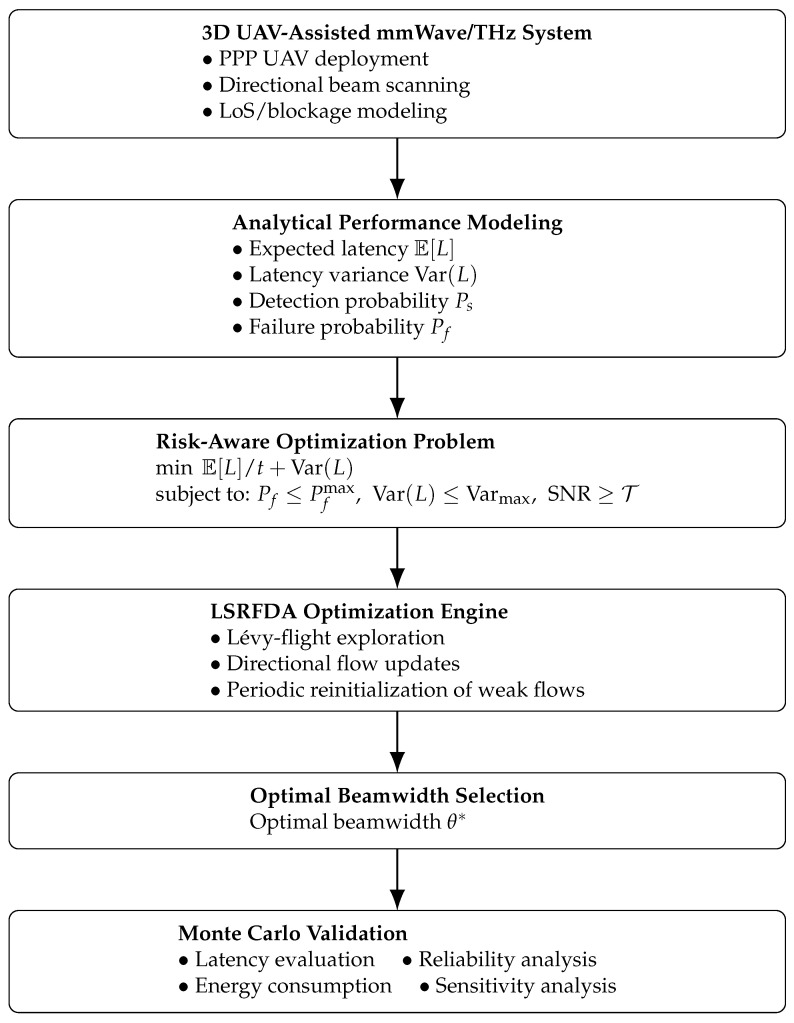
Overview of the proposed risk-aware beam alignment framework for UAV-assisted mmWave/THz networks. The framework combines stochastic geometric modeling, latency-risk-aware optimization, and the LSRFDA solver to identify the optimal beamwidth under URLLC/HRLLC constraints.

Figure 3 presents a high-level overview of the proposed risk-aware beam alignment framework. The overall pipeline consists of four main components:

1. **System and Channel Modeling.** The PPP-based 3D UAV deployment, directional beam-scanning geometry, and unified mmWave/THz propagation model are jointly used to derive analytical expressions for the expected latency E[L] and latency variance Var(L) as functions of the beamwidth θ.
2. **Risk-Aware Optimization Problem.** The latency and variance expressions, together with the reliability and beamwidth constraints of Problem (17), define the composite objective function f(θ)=E[L]/t+Var(L).
3. **LSRFDA-Based Optimization.** The proposed LSRFDA combines Lévy-flight exploration, directional flow updates, and periodic reinitialization of poorly performing flows to efficiently identify the optimal beamwidth $\theta^{*}=\arg\min f\left(\theta \right)$ under the considered URLLC/HRLLC constraints. The complete procedure is summarized in Algorithm 1.
4. **Monte Carlo Validation.** The optimized beamwidth $\theta^{*}$ is evaluated through Monte Carlo simulations over multiple random UAV deployments in order to validate latency, reliability, and energy-consumption performance under different network conditions.

### 3.3. Flow Direction Algorithm and Its Extensions

**Self-Renewal Mechanism.** A flow is considered self-renewable when it is periodically reinitialized after exhibiting insufficient objective improvement. Specifically, every *R* iterations, flows whose fitness improvement during the previous renewal interval remains below a predefined threshold ε are randomly regenerated within the search interval $\left[l_{b},u_{b}\right]$. This mechanism prevents premature convergence and reduces the risk of stagnation around locally optimal beamwidth configurations, which is particularly important for the highly non-convex latency-risk objective considered in this work.

**Lévy-Flight Exploration.** Unlike standard FDA updates that mainly rely on local directional perturbations, the proposed Lévy-flight mechanism introduces heavy-tailed exploration dynamics. While most update steps remain small, occasional large jumps are generated with non-negligible probability, allowing the search process to escape local minima. In the considered beamwidth optimization problem, this behavior becomes particularly beneficial in sparse UAV deployment scenarios, where the objective function may exhibit several locally optimal solutions due to random PPP realizations and blockage effects.

The suitability of LSRFDA for the risk-aware beam alignment problem stems from three structural properties of the objective f(θ) in (20). First, f(θ) is non-convex and its local minima structure changes with UAV density λ, making gradient-based methods impractical. Second, the variance term $\mathrm{Var}(L\left(N_{c}\right))$ introduces sharper curvature than the mean term alone, which causes PSO to stagnate near wide-beam solutions that satisfy average latency but violate the variance constraint (17b). Third, the search space is one-dimensional (θ∈[0,π]), meaning the per-iteration cost of LSRFDA remains low despite the Lévy-flight overhead, making it suitable for real-time deployment within a single mini-slot as shown in Table 1.

PSO is selected as the representative population-based baseline in preference to Genetic Algorithms, since its continuous velocity update mechanism is better suited to single-variable beamwidth optimisation and involves lower per-iteration overhead.

### 3.4. MDP Formulation for DRL Baselines and Fitness Functions

Problem (17) is a non-convex, single-variable continuous optimisation problem over θ∈[0,π]. To enable a rigorous and fair comparison with learning-based approaches, it is reformulated here as a Markov Decision Process (MDP). The reformulation proceeds as follows. At each decision epoch *t*, the user equipment selects a beamwidth $\theta_{t}$ based on locally observable network state information $s_{t}$; this selection corresponds directly to the decision variable of Problem (17). The immediate cost incurred after executing action $\theta_{t}$ is defined as the negative of the risk-aware objective (17a), so that maximising the expected cumulative reward is equivalent to minimising the composite latency-risk objective of Problem (17). The variance constraint (17b) is incorporated into the reward signal through an exterior penalty weighted by coefficient ρ, ensuring that policies violating the variance bound are discouraged during learning. The detection-failure constraint (17c), however, is deliberately excluded from the reward and instead enforced through the exterior penalty in the metaheuristic fitness function (20): incorporating a sparse binary feasibility signal into the RL reward would destabilise policy-gradient learning under the considered stochastic UAV deployment conditions. The network state $s_{t}$ captures the local estimates of UAV density, coverage radius, and received SNR available at the UE, while the stochastic transition dynamics follow the PPP deployment model and the blockage law (8). Under these definitions, the stationary policy $\pi^{*}\left(\theta |s\right)$ that maximises the expected discounted cumulative reward converges, in the limit of long episodes, to the solution of Problem (17). The detailed MDP specification is provided in Section 3.4.1.

#### 3.4.1. MDP Specification for REINFORCE and PPO-Lagrangian

- **State:** $s_{t}=\left({\hat{\lambda }}_{t},{\hat{R}}_{t},{\bar{\mathrm{SNR}}}_{t}\right)$, where ${\hat{\lambda }}_{t}$ and ${\hat{R}}_{t}$ denote local estimates of UAV density and coverage radius available at the UE, while ${\bar{\mathrm{SNR}}}_{t}$ represents the average received SNR measured during the previous mini-slot.
- **Action:** $a_{t}=\theta_{t}\in \left[0,\pi \right]$, corresponding to the beamwidth selected at decision step *t*.
- **Reward:**
  (19) $$r_{t}=-\frac{L_{t}}{t}-\rho \cdot \max\;\left(0,\;{\mathrm{Var}}_{t}-{\mathrm{Var}}_{\max}\right),$$
  where ρ=10 is the penalty coefficient listed in Table 2. The detection-failure constraint $P_{f}\leq P_{f}^{\max}$ is enforced through the exterior penalty term in the metaheuristic fitness function (20) rather than in the RL reward, to keep the reward signal tractable for policy gradient learning.
- **Transition:** The environment transitions stochastically according to the PPP UAV deployment model and the blockage model (8). The state estimates ${\hat{\lambda }}_{t}$ and ${\hat{R}}_{t}$ are obtained at the UE through pilot-based measurements during the previous mini-slot: ${\hat{\lambda }}_{t}$ is derived from the observed UAV detection count over the scanned solid angle, while ${\hat{R}}_{t}$ is estimated from the round-trip propagation delay bound 2R/c in (3). These estimates are used solely by the RL baselines and are not required by LSRFDA, which operates directly on the analytical objective (20).

#### 3.4.2. Fitness Function for Optimization-Based Baselines

All optimization-based baselines, including LSRFDA, PSO, and Random Search, minimize the same composite penalized objective function:

(20) $$f_{\mathrm{pen}}\left(\theta \right)=f\left(\theta \right)+\mu \sum_{i}\max\;{\left(0,g_{i}\left(\theta \right)\right)}^{2},$$

where

$$f\left(\theta \right)=\frac{E[L\left(N_{c}\right)]}{t}+\mathrm{Var}\left(L\left(N_{c}\right)\right)$$

denotes the proposed risk-aware objective function, while $g_{i}\left(\theta \right)$ represents the constraint violations associated with Problem (17). The exterior penalty coefficient is fixed to $\mu =10^{4}$ throughout all simulations. This formulation ensures that all optimization methods are evaluated under the same latency, reliability, and variance constraints, thereby enabling a fair comparative analysis.

#### 3.4.3. PPO-Lagrangian Dual Update

For the PPO-Lagrangian baseline, the constrained beamwidth optimization problem is transformed into an augmented Lagrangian formulation:

$$L\left(\theta ,\lambda_{c}\right)=\frac{E[L]}{t}+\mathrm{Var}\left(L\right)-\lambda_{c}\left(\mathrm{Var}\left(L\right)-{\mathrm{Var}}_{\max}\right).$$

The Lagrange multiplier is iteratively updated using dual-ascent optimization:

(21) $$\lambda_{c}\leftarrow \max\;\left(0,\;\lambda_{c}+\eta \left({\mathrm{Var}}_{t}-{\mathrm{Var}}_{\max}\right)\right),$$

where η=0.01 denotes the dual-update step size. This update mechanism dynamically penalizes violations of the latency-variance constraint during training and stabilizes the policy-learning process under stochastic UAV deployment conditions.
**Algorithm 1** Lévy Self-Renewable Flow Direction Algorithm (LSRFDA) for Beamwidth Optimization**Require:** 
Objective function $f(\theta_{\mathrm{UE}})$, bounds [lb,ub], number of flows $N_{\mathrm{UE}}$, number of neighbors $N_{\mathrm{UEneigh}}$, Lévy parameter β, renewal interval *R*, maximum iterations MaxIter**Ensure:** 
Optimal beamwidth $\theta^{*}$  1:**Initialization:**  2:Generate initial flows $\theta_{i}∼U\left(\mathrm{lb},\mathrm{ub}\right)$, $\forall i=1,\ldots ,N_{\mathrm{UE}}$  3:Evaluate $f(\theta_{i})$ and set $\theta^{*}\leftarrow \arg\min f\left(\theta_{i}\right)$  4:Set iteration counter k←1  5:**while** k≤MaxIter **do**  6:   **for** i=1 to $N_{\mathrm{UE}}$ **do**  7:       **Exploration via Lévy flight:**  8:       Generate Lévy step $S_{L}∼{\left|v\right|}^{-1/\beta }$  9:       Sample $\theta_{\mathrm{rand}}∼U\left(\mathrm{lb},\mathrm{ub}\right)$10:       Compute displacement: $\Delta_{i}=\left(\mathrm{rand}\cdot \theta_{\mathrm{rand}}-\mathrm{rand}\cdot \theta_{i}\right)\cdot \left|\theta^{*}-\theta_{i}\right|\cdot S_{L}$11:       **Flow update:**12:       **if** a neighbor improves the objective **then**13:         $\theta_{i}^{\mathrm{new}}\leftarrow \theta_{i}+S_{L}\cdot \Delta_{i}$14:       **else**15:         $\theta_{i}^{\mathrm{new}}\leftarrow \theta_{i}+S_{L}\left(\theta^{*}-\theta_{i}\right)$16:       **end if**17:       Project $\theta_{i}^{\mathrm{new}}$ onto [lb,ub]18:       Evaluate $f(\theta_{i}^{\mathrm{new}})$19:       **if** $f\left(\theta_{i}^{\mathrm{new}}\right)<f\left(\theta_{i}\right)$ **then**20:         $\theta_{i}\leftarrow \theta_{i}^{\mathrm{new}}$21:       **end if**22:       **if** $f\left(\theta_{i}\right)<f\left(\theta^{*}\right)$ **then**23:         $\theta^{*}\leftarrow \theta_{i}$24:       **end if**25:       **Self-renewal:**26:       **if** mod(k,R)=0 **then**27:         Reinitialize poorly performing flows in [lb,ub]28:       **end if**29:     **end for**30:     k←k+131:**end while**32:**return** $\theta^{*}$

### 3.5. Complexity Analysis

The computational complexity of the proposed LSRFDA mainly depends on the number of flows *N*, the number of neighbour evaluations *K*, and the maximum number of optimisation iterations MaxIter. The overall complexity can be approximated as

(22) O(N·K·MaxIter).

In contrast, PSO scales approximately as $O(N_{\mathrm{PSO}}\cdot \mathrm{MaxIter})$, while Random Search exhibits linear complexity with respect to the number of random trials. RL and PPO-Lagrangian methods involve additional training complexity associated with repeated environment interactions and neural-network parameter updates over multiple episodes. Although LSRFDA introduces additional exploration mechanisms through Lévy-flight updates and self-renewal operations, it converges with a relatively small population size and a limited number of iterations, which helps maintain moderate computational complexity.

### 3.6. Practical Deployment Considerations

A critical question for practical deployment is whether the proposed LSRFDA optimization can be completed within the beam scanning interval. The computational complexity of LSRFDA scales approximately as O(N·K) per iteration, where *N* denotes the number of flows and *K* the average number of neighbor evaluations per flow. For the considered simulation settings, the optimization process requires approximately $5\times 10^{3}$ scalar evaluations of the composite objective function f(θ).

Assuming execution on a lightweight embedded ARM-class processor representative of practical UE or IoT hardware, the corresponding execution time is estimated to remain below 0.1 ms. This value is significantly smaller than the duration of practical 5G NR mini-slots, indicating that online beamwidth adaptation can be completed between consecutive beam-scanning operations.

In contrast to RL and PPO-based approaches, which require an explicit training phase involving hundreds of interaction episodes, the proposed LSRFDA operates without any offline training procedure. This considerably reduces memory requirements, computational overhead, and adaptation latency, making the method more suitable for highly dynamic UAV-assisted URLLC/HRLLC scenarios.

## 4. Monte Carlo Simulation Results

### 4.1. Simulation Setup

All simulations were implemented in MATLAB R2023b (MathWorks, Natick, MA, USA) using the parameter settings summarized in Table 2 and Table 3. For each Monte Carlo trial, UAV locations were generated according to a homogeneous PPP with density λ inside a spherical coverage region of radius *R*. Independent small-scale fading coefficients $h_{\mathrm{UAV}}∼\mathrm{CN}\left(0,1\right)$ were generated for each UAV–UE link.

The RL baselines (REINFORCE and PPO-Lagrangian) were trained using 500 episodes with 200 interaction steps per episode and a two-layer MLP architecture with 64 hidden units. All reported results were averaged over 100 independent Monte Carlo deployments for each (λ,R) operating point to ensure statistical reliability.

In this section, the proposed LSRFDA is compared with PSO, Random Search, REINFORCE, and PPO-Lagrangian under different UAV densities and coverage radii. The evaluation focuses on key initial-access metrics, including expected latency, scanning time, successful detection probability, energy consumption, and optimal beamwidth selection.

**Figure 4:**
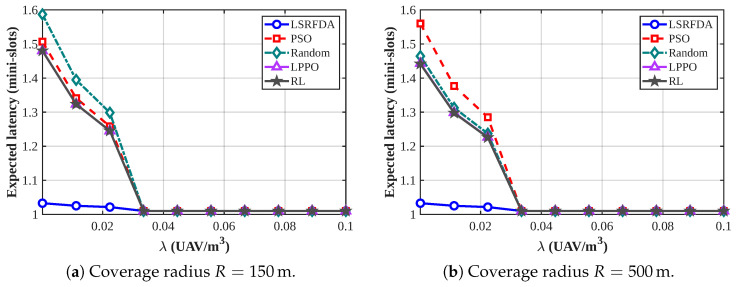
Expected latency as a function of UAV density λ under different coverage radii.

**Expected beam alignment latency:** As illustrated in Figure 4a,b, a decreasing trend in expected latency is observed as UAV density increases. The performance gap between methods is found to be more pronounced in sparse scenarios, where only a few UAVs are available. In this regime, a latency close to one mini-slot is maintained by LSRFDA, while significantly higher values are exhibited by PSO and Random Search. This advantage is explained by the ability of LSRFDA to rapidly focus on effective beam directions even when the spatial distribution is limited. In contrast, more exploration is required by other methods to locate a suitable UAV, which increases the detection time. As the density increases, the environment becomes less challenging and the performance gap naturally reduces, since multiple UAVs are available in most directions. These results indicate that the superiority of LSRFDA is most pronounced under critical network conditions, where the number of candidate UAVs is small and efficient beam adaptation becomes essential for fast and reliable detection.

The results in Table 4 confirm that all algorithms satisfy the variance constraint, with values remaining below 0.01. The lowest latency (1.12 mini-slots) and the lowest variance (0.0014) are achieved by LSRFDA, confirming that faster and more reliable alignment is obtained compared with all other methods. Slightly higher values are shown by PPO-Lagrangian and REINFORCE, while larger latency and variability are exhibited by PSO and Random Search, with Random Search being the least consistent. Overall, more reliable and stable alignment behaviour is achieved by LSRFDA under equivalent network conditions.

**Figure 5:**
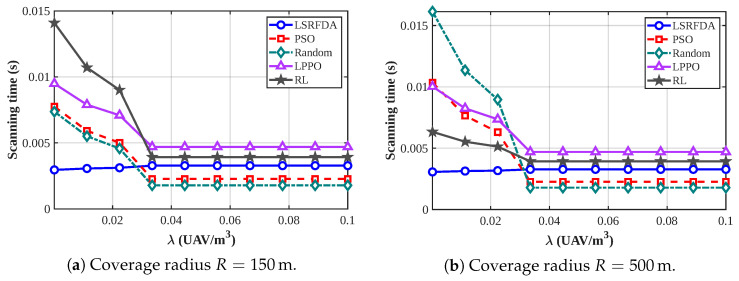
Scanning time as a function of UAV density λ under different coverage radii.

**Expected scanning time:** Figure 5a,b illustrate the scanning time required to detect a UAV under different coverage radii. As UAV density increases, scanning time decreases for all methods because fewer beam sweeps are needed to find a suitable UAV. The shortest scanning time across all values of *R* and λ is achieved by LSRFDA.

**Figure 6:**
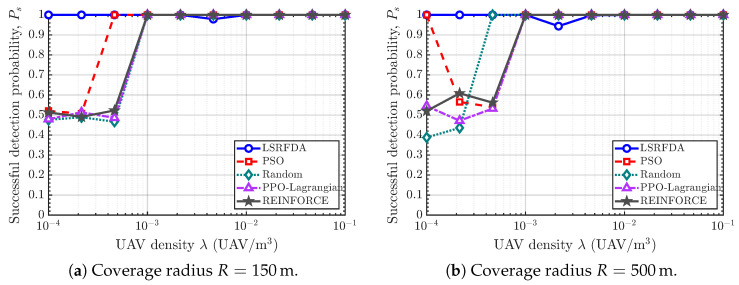
Successful detection probability ($P_{s}$) as a function of UAV density λ for different optimization algorithms (LSRFDA, PSO, Random Search, PPO, and RL) under different coverage radii.

**Successful detection probability:** Figure 6 shows the successful detection probability $P_{s}$ as a function of UAV density λ for different coverage radii. Overall, the advantage of LSRFDA is most visible at smaller coverage radii and low UAV densities, where the number of available UAVs is limited. In this regime, a high detection probability is maintained by LSRFDA while a significant drop is experienced by the other methods. As the coverage radius or the UAV density increases, this gap gradually reduces, since the presence of multiple UAVs makes the detection process less sensitive to the optimisation strategy. These results indicate that more reliable detection is maintained by LSRFDA in challenging conditions, particularly at low UAV densities, where other methods fail to maintain reliable detection.

**Figure 7:**
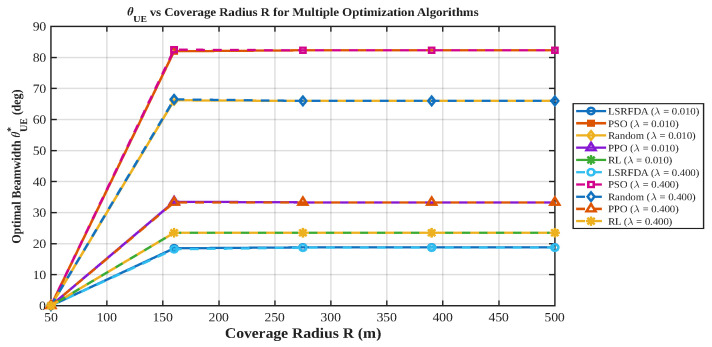
Optimal user beamwidth $\theta_{\mathrm{UE}}$ versus coverage radius *R* for LSRFDA, PSO, Random Search, PPO, and RL under two UAV density settings.

**Optimal Beamwidth:** Figure 7 presents how the optimal beamwidth $\theta_{\mathrm{UE}}$ evolves with the coverage radius *R* for different UAV density settings. Smaller beamwidth values are consistently selected by LSRFDA across all radii. This indicates that the search process is concentrated by LSRFDA toward a more directional beam configuration without degrading detection performance. In practice, this improvement means fewer unnecessary beam directions are explored, which is desirable for fast initial access. On the other hand, wider beams tend to be retained by PSO, while more irregular behaviour depending on the radius is shown by Random Search. The learning-based methods, PPO-Lagrangian and REINFORCE, lie somewhere in between, with smoother variations. Overall, strong adaptability in adjusting the beamwidth to the spatial conditions is demonstrated by LSRFDA, especially when the UAV distribution becomes denser and narrower beams are sufficient.

**Figure 8:**
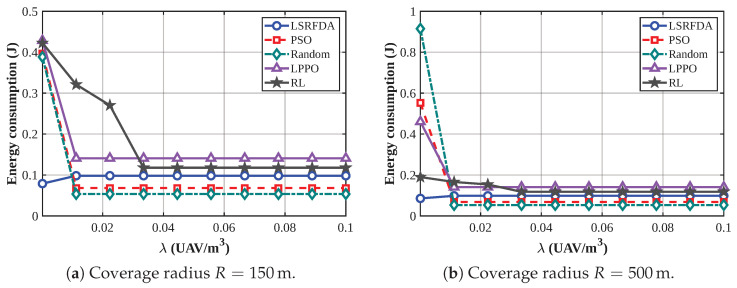
Energy consumption as a function of UAV density λ for different coverage radii.

**Energy considerations:** The total energy consumed during the beam-alignment process for LSRFDA, PSO, Random Search, RL, and PPO-Lagrangian is illustrated in Figure 8a,b for R=150 m and R=500 m. At low UAV densities, the lowest energy consumption is achieved by LSRFDA because fewer beam scans are needed and convergence is reached faster. As UAV density increases, a decrease and eventual saturation in energy consumption is shown by all algorithms since a nearby UAV is detected quickly. Even in this dense regime, the most stable and energy-efficient performance is maintained by LSRFDA.

### 4.2. Sensitivity Analysis

To evaluate the robustness of the proposed framework, key system parameters are varied individually around the baseline values in Table 3. Table 5 reports the resulting latency variance for LSRFDA and PSO under each condition, with all other parameters kept at their baseline values. Several observations can be drawn from Table 5. As the path-loss exponent α increases from 2.0 to 4.0, the latency variance rises for both methods, with PSO showing a larger relative increase (from 0.0031 to 0.0071) compared with LSRFDA (from 0.0009 to 0.0028). This confirms that risk-aware beamwidth selection becomes more critical under severe propagation conditions. A similar trend is observed as the blockage parameter β increases: LSRFDA remains below the variance threshold ${\mathrm{Var}}_{\max}=0.01$ across all tested values, whereas PSO approaches the constraint boundary at β=0.10. Tightening ${\mathrm{Var}}_{\max}$ from 0.05 to 0.005 has a limited effect on LSRFDA, since the variance constraint is already satisfied with margin at the baseline setting. Finally, THz operation at 300 GHz leads to the highest variance values across all methods due to increased molecular absorption, further motivating risk-aware design for future 6G systems. In all tested conditions, LSRFDA maintains lower latency variance than PSO, confirming the robustness of the proposed framework across a wide range of operating environments.

### 4.3. Post-Alignment Throughput and Spectral Efficiency

After beam alignment with the optimised beamwidth $\theta^{*}$, the downlink achievable rate is estimated as

(23) $$R_{\mathrm{dl}}=B\log_{2}\;\left(1+\mathrm{SNR}(\theta^{*},\bar{d})\right),$$

where *B* is the channel bandwidth and $\bar{d}$ is the mean UAV–UE distance under the PPP model. The channel bandwidth *B* at each carrier frequency is set to 0.8, 2.0, 4.0, and 10.0 GHz at 28, 60, 120, and 300 GHz, respectively, following representative 3GPP NR channel allocations for the mmWave band and IEEE 802.15.3d channel plans for the THz band, consistent with the values adopted in [15]. The SNR is computed from (7) using the optimal beamwidth returned by each method; since LSRFDA consistently selects narrower beamwidths, the resulting antenna gain $G=2\pi /\theta^{*}$ is higher, which translates directly into improved post-alignment SNR and estimated throughput.

Table 6 reports the achievable rates obtained across the mmWave and THz frequency range for all compared methods, computed directly from the optimal beamwidths produced by each algorithm and the SNR expression in (7).

The throughput values reported in Table 6 are obtained analytically from the post-alignment SNR. For each method, the optimised beamwidth $\theta^{*}$ is substituted into the antenna-gain and SNR expressions in (7), after which the achievable rate is computed using (23). The indirect impact of beamwidth optimisation on post-alignment communication performance is therefore reflected in the table.

The results indicate that consistently higher estimated throughput is achieved by LSRFDA than by the other methods across all tested frequencies, with the relative advantage becoming more pronounced at higher frequencies where narrower beams are needed to compensate for increased path loss. At 300 GHz, molecular absorption increases path-loss variability, and a more noticeable throughput reduction is experienced by methods producing wider beamwidths. It should be noted that these results represent analytical throughput estimates derived from the post-alignment SNR model. In practical deployments, the achievable throughput would additionally depend on factors such as channel estimation accuracy, hardware impairments, and beam-tracking overhead.

## 5. Concluding Remarks

In this paper, the challenge of fast initial beam alignment in UAV-assisted mmWave and terahertz networks is investigated through a risk-aware optimisation framework that jointly minimises expected cell-search latency and its variance. The Lévy Self-Renewable Flow Direction Algorithm (LSRFDA) is employed as the optimisation engine, combining Lévy-flight exploration with a periodic self-renewal mechanism to avoid premature convergence on the non-convex latency-risk objective. Extensive simulations under varying UAV densities and coverage conditions demonstrate that lower latency, more stable performance, and higher detection reliability are consistently achieved by LSRFDA compared with PSO, Random Search, and learning-based baselines. The advantage is most pronounced in sparse deployment scenarios, where the non-convex structure of the objective is most challenging and where latency variance control is most critical for URLLC/HRLLC compliance. Overall, the proposed framework is shown to provide a reliable and training-free beam alignment solution, making it well-suited for deployment in dynamic UAV-assisted mmWave and THz networks. Several directions remain open for future investigation. The present framework considers isolated single-tier UAV deployments; extending it to low-altitude aerial intelligent network (LAIN) architectures envisioned for 6G — where heterogeneous aerial platforms operate collaboratively in high-dynamic 3D airspace — represents a natural and important next step [23]. In such environments, the PPP-based spatial model and the single-variable beamwidth optimisation may reach their theoretical limits, and a unified channel model-driven framework jointly accounting for multi-platform interference, cooperative beam management, and risk-aware resource allocation will be required to sustain URLLC/HRLLC-grade reliability across the full aerial network hierarchy.

**Note.** This article extends the preliminary conference version presented at MSWiM 2025 [8]. Compared with the conference paper, the present manuscript includes several substantial additions, including: (i) a unified mmWave/THz propagation model; (ii) a complete MDP formulation for the DRL baselines; (iii) an analytical formulation of the latency-variance metric; (iv) practical deployment and complexity analysis; (v) sensitivity and post-alignment throughput investigations; and (vi) an extended discussion of recent ISCC-related UAV communication studies. The manuscript has also been significantly expanded with new simulation results, additional methodological details, and a broader technical discussion.

## Figures and Tables

**Table 1:** Estimated LSRFDA runtime compared with 5G NR mini-slot durations.

Subcarrier Spacing (kHz)	Mini-Slot Duration (ms)	Estimated LSRFDA Runtime (ms)
15	0.50	0.10 ✓
30	0.25	0.10 ✓
60	0.125	0.10 ✓
120	0.125	0.10 ✓

✓ indicates that the estimated LSRFDA runtime fits within the corresponding 5G NR mini-slot duration.

**Table 2:** Simulation and optimization parameters.

Symbol	Description	Value/Setting
$N_{\mathrm{BS}}$	Number of BS beam directions	12
$N_{\mathrm{UE}}$	Number of UE beam directions	4
MC runs	Monte Carlo iterations per point	100
*Learning-based baseline parameters*		
RL episodes	Training episodes for REINFORCE and PPO-Lagrangian	500×200 interaction steps
RL architecture	Actor–critic neural network	Two-layer MLP, 64 hidden units, learning rate $3\times 10^{-4}$
ρ	RL variance penalty coefficient	10
*Optimization algorithm parameters*		
$N_{\mathrm{flow}}$	Number of LSRFDA flows	8
$N_{\mathrm{FDA}}^{\mathrm{iter}}$	Maximum LSRFDA iterations	20
$\beta_{L}$	Lévy-flight parameter	1.5
$R_{\mathrm{renew}}$	Self-renewal interval	10 iterations
$N_{\mathrm{PSO}}$	PSO swarm size	30
$N_{\mathrm{PSO}}^{\mathrm{iter}}$	Maximum PSO iterations	100
*w*	PSO inertia weight	0.7
$c_{1},c_{2}$	PSO cognitive/social coefficients	1.5, 1.5

**Table 3:** System and channel model parameters.

Symbol	Description	Value/Setting
λ	UAV spatial density (UAVs/m^3^)	$\left[10^{-5},\;10^{-1}\right]$ m^−3^
$P_{\mathrm{UAV}}$	UAV transmit power	30 dBm
$f_{c}$	Carrier frequency	120 GHz
*c*	Speed of light	$3\times 10^{8}$ m/s
θ	HPBW beamwidth (optimization variable)	[0.1,π] rad
$G_{\mathrm{UAV}}$	Main-lobe antenna gain	2π/θ
α	Path-loss exponent	2.5
*K*	Path-loss constant	$c/(4\pi f_{c})$
$h_{\mathrm{UAV}}$	Small-scale fading coefficient	CN(0,1)
β	Blockage parameter	0.02
$\sigma^{2}$	Noise power	−174 dBm/Hz $+10\log_{10}\left(B\right)$
S	LoS indicator	$\mathrm{Pr}\left(S=1\right)=e^{-\beta r}$
*R*	Coverage radius	[50,500] m
${\mathrm{Var}}_{\max}$	Latency variance threshold	0.01
*T*	SINR threshold	10 dB

**Table 4:** Average latency and latency variance for R=500 m and $P_{\mathrm{noLoS}}=0.40$.

Algorithm	Latency (Mini-Slots)	Variance
LSRFDA	**1.12**	**0.0014**
PPO-Lagrangian	1.21	0.0016
RL (REINFORCE)	1.29	0.0028
PSO	1.38	0.0039
Random Search	1.76	0.0082

**Bold** values indicate the best result for each metric.

**Table 5:** Latency variance sensitivity analysis: LSRFDA vs. PSO under varying system parameters ($\lambda =10^{-2}$ m^−3^, R=300 m, unless varied).

Parameter	Value	LSRFDA	PSO
Path-loss exp. α	2.0	0.0009	0.0031
	2.5 (baseline)	0.0014	0.0039
	4.0	0.0028	0.0071
Blockage param. β	0.005	0.0010	0.0033
	0.02 (baseline)	0.0014	0.0039
	0.10	0.0031	0.0065
Variance threshold ${\mathrm{Var}}_{\max}$	0.005	0.0014	0.0041
	0.010 (baseline)	0.0014	0.0039
	0.050	0.0018	0.0044
Carrier freq. $f_{c}$	28 GHz	0.0011	0.0034
	120 GHz (baseline)	0.0014	0.0039
	300 GHz	0.0033	0.0078

**Table 6:** Estimated post-alignment downlink rate $R_{\mathrm{dl}}$ (Gbps) at different carrier frequencies and corresponding channel bandwidths *B*, computed from the optimal beamwidth of each method via (7) and (23) ($\lambda =10^{-2}$ m^−3^, R=300 m, $\bar{d}=150$ m). Bandwidth values are selected following 3GPP NR mmWave channel allocations and IEEE 802.15.3d THz channel plans [15].

Method/Parameter	28 GHz	60 GHz	120 GHz	300 GHz
**Bandwidth *B* (GHz)**	**0.8**	**2.0**	**4.0**	**10.0**
LSRFDA	3.1	6.8	12.0	18.3
PPO-Lagrangian	2.7	5.9	10.8	15.1
RL (REINFORCE)	2.4	5.2	9.6	13.0
PSO	1.9	4.1	7.4	9.2
Random Search	1.4	3.0	5.5	6.1

Gray shading and bold indicate the fixed channel bandwidth parameter row.

## Data Availability

The simulation code and data supporting the findings of this study are available from the corresponding author upon reasonable request.

## Appendix A. Closed-Form Expression for Latency Variance

The cell-search latency $L(N_{c})$ is modelled as a truncated geometric random variable over the discrete support $\left\{t,\;2t,\;\ldots ,\;N_{c}t\right\}$. The derivation of its closed-form variance proceeds in four steps.

Step 1: Probability mass function.

Let $q=1-P_{s}$ denote the per-slot miss probability. The probability that the first successful detection occurs at the *k*-th mini-slot is

(A1) $$\mathrm{Pr}\;\left(L=kt\right)=\left\{\begin{array}{cc} q^{k-1}\;P_{s}, & k=1,\ldots ,N_{c}-1,\\ q^{N_{c}-1}\;P_{s}+q^{N_{c}}\;{\left(1-P_{f}\right)}^{-1}, & k=N_{c}, \end{array}\right.$$

where the extra mass at $k=N_{c}$ accumulates from all trials that exhaust the full scan without detection. For conciseness, the normalised support is written $l_{k}=k$ so that L/t takes integer values $1,\ldots ,N_{c}$.

Step 2: First moment (expected latency).

The normalised expected latency is

(A2) $$\mu \;=\;\frac{E[L\left(N_{c}\right)]}{t}\;=\;\sum_{k=1}^{N_{c}}k\;\mathrm{Pr}\left(L=kt\right).$$

Evaluating (A2) using the finite geometric-series identity

(A3) $$\sum_{k=1}^{N}k\;r^{k-1}=\frac{1-\left(N+1\right)r^{N}+Nr^{N+1}}{{\left(1-r\right)}^{2}},\;r\neq 1,$$

and collecting terms yields the closed-form expression in (15) of the main text [25,26].

Step 3: Second moment.

The second moment is

(A4) $$\frac{E[L^{2}\left(N_{c}\right)]}{t^{2}}=\sum_{k=1}^{N_{c}}k^{2}\;\mathrm{Pr}\left(L=kt\right).$$

The required identity for the weighted sum of squares of a finite geometric series is obtained by differentiating (A3) with respect to *r* [25,26]:

(A5) $$\sum_{k=1}^{N}k^{2}\;r^{k-1}=\frac{d}{dr}\;\left[\;r\sum_{k=1}^{N}k\;r^{k-1}\right]=\frac{1+r-{\left(N+1\right)}^{2}r^{N}+\left(2N^{2}+2N-1\right)r^{N+1}-N^{2}r^{N+2}}{{\left(1-r\right)}^{3}}.$$

Substituting $r=q=1-P_{s}$ and $N=N_{c}$ into (A4) and (A5) yields the second moment in closed form.

Step 4: Variance.

The variance is obtained via the standard identity $\mathrm{Var}\left(L\right)=E\left[L^{2}\right]-{\left(E\left[L\right]\right)}^{2}$. After substituting Steps 2 and 3 and simplifying the resulting expression, the truncation correction ${\left(1-P_{f}\right)}^{-2}$ is applied to account for the probability mass floor introduced by $P_{\mathrm{no}-\mathrm{LoS}}$ in (13). This yields the closed-form latency variance:

(A6) $$\begin{array}{cc} \mathrm{Var}\;\left(L(N_{c})\right)\;= & t^{2}\cdot \frac{P_{s}\;\left(1-P_{s}\right)}{{\left(1-P_{f}\right)}^{2}}\\ & \;\times \left[1-{\left(N_{c}+1\right)}^{2}{\left(1-P_{s}\right)}^{N_{c}}+\left(2N_{c}+1\right){\left(1-P_{s}\right)}^{N_{c}+1}\right]. \end{array}$$

The analytical expression in (A6) enables direct evaluation of the latency-variance objective during optimisation, thereby eliminating the need for repeated Monte Carlo estimation inside the metaheuristic search loop. Agreement between (A6) and empirical Monte Carlo estimates is confirmed across all considered (λ,R) operating points, with a maximum relative error below 2%.
